# The Atomic Rearrangement of GaN-Based Multiple Quantum Wells in H_2_/NH_3_ Mixed Gas for Improving Structural and Optical Properties

**DOI:** 10.1186/s11671-021-03618-8

**Published:** 2021-11-02

**Authors:** Yuhao Ben, Feng Liang, Degang Zhao, Jing Yang, Zongshun Liu, Ping Chen

**Affiliations:** 1 State Key Laboratory of Integrated Optoelectronics, Institute of Semiconductors, Chinese Academy of Science, Beijing, 100083 China; 2 College of Materials Science and Opto-Electronic Technology, University of Chinese Academy of Sciences, Beijing, 100049 China; 3 Center of Materials Science and Optoelectronics Engineering, University of Chinese Academy of Sciences, Beijing, 100049 China

**Keywords:** Atomic rearrangement, Hydrogen treatment, GaN-based MQWs

## Abstract

In this work, three GaN-based multiple quantum well (MQW) samples are grown to investigate the growth techniques of high-quality MQWs at low temperature (750 °C). Instead of conventional temperature ramp-up process, H_2_/NH_3_ gas mixture was introduced during the interruption after the growth of InGaN well layers. The influence of hydrogen flux was investigated. The cross-sectional images of MQW via transmission electron microscope show that a significant atomic rearrangement process happens during the hydrogen treatment. Both sharp interfaces of MQW and homogeneous indium distribution are achieved when a proper proportion of hydrogen was used. Moreover, the luminescence efficiency is improved strongly due to suppressed non-radiative recombination process and a better homogeneity of MQWs. Such kind of atomic rearrangement process is mainly caused by the larger diffusion rate of gallium and indium adatoms in H_2_/NH_3_ mixed gas, which leads to a lower potential barrier energy to achieve thermodynamic steady state. However, when excessive hydrogen flux is introduced, the MQW will be partly damaged, and the luminescence performance will deteriorate.

## Introduction

In recent years, InGaN/GaN multiple quantum wells (MQWs) have been extensively investigated due to its excellent application potentials in highly efficient optoelectronic devices operating in the whole visible spectral regions [1–5]. However, it is still challenging to grow high-quality MQW with higher indium composition for pure blue and green light emitting diodes (LEDs) and lasers diodes (LDs) via metal organic chemical vapor deposition (MOCVD). There are two aspects increasing the difficulty of the growth of high-quality MQWs. On one hand, the indium incorporation is difficult at high temperature due to weak binding energy to the surface [6]. Thus, the growth temperature of InGaN well layers is normally below 800 °C [7]. But at such low temperature, the diffusion rate of gallium will be restricted strongly, leading to three-dimensional growth of GaN barrier layers and poor surface morphology of MQW region [8, 9]. On the other hand, the large lattice and thermal mismatch between InN and GaN will lead to phase segregation [10–12] and compositional grading of indium [13, 14], resulting a poor homogeneity of indium distribution in MQW region [15, 16].

To solve these problems, various growth techniques have been employed to strive for sharp interfaces in the MQW and a homogeneous distribution of indium composition. Growth of barrier layers at a higher temperature [17, 18], temperature ramp-up process after the growth of QWs [19, 20], the interruption of growth between quantum barriers (QBs) and QWs [21, 22], and growth of barriers in hydrogen atmosphere [23, 24] are known to be effective for the quality improvement of MQWs. However, in most techniques, the temperature ramp-up process is necessary, which will hinder the indium incorporation and causes thermal degradation of MQWs with higher indium content. When the emission wavelength comes to blue and green range, a higher indium content in MQWs is required, which will be hindered by the temperature ramp-up process. Therefore, new techniques without temperature ramp-up process need further investigation. In this case, introducing hydrogen in the growth of MQWs is a feasible alternative. In previous work, hydrogen has been introduced into the growth process of GaN barrier layers [9], helping increase the diffusion rate of gallium adatoms and achieve a better surface morphology of MQW region [23]. But for the growth of InGaN well layers, researchers found that even a small amount hydrogen will strongly deteriorate the indium incorporation [6, 25]. As a result, hydrogen is not widely used in the growth of InGaN epilayers [18, 21].

In this work, instead of high flux hydrogen, H_2_/NH_3_ mixed gas was introduced during the interruption after the growth of InGaN well layers. To protect well layers, an extra thin GaN cap layer was deposited before the introduction of maxed gas. Three MQW samples are grown completely at lower temperature (750 °C) without temperature ramp-up process. The properties of MQW were characterized by the transmission electron microscope (TEM), high-resolution X-ray diffraction (HRXRD), temperature-dependent photoluminescence spectra and laser scanning confocal microscopy. An obvious atomic rearrangement process of MQW has been observed when using appropriate hydrogen flow rate. Both sharp interfaces and homogeneous distribution of indium composition are achieved. As a result, a much higher luminescence efficiency has been achieved. Our work indicates that high-quality of MQW regions grown at lower temperature can be achieved by using hydrogen treatment under NH3 atmosphere, which may be helpful to the fabrication of higher indium content blue and green LDs/LEDs.

## Experimental Process

Three InGaN/(In)GaN MQW samples, named samples A, B and C, were grown on c-plane sapphire substrate by a Thomas Swan 3 × 2 in. close-coupled showerhead reactor MOCVD. During the epitaxial growth process, the triethylgallium (TEGa), trimethylindium (TMIn) and ammonia (NH_3_) were used as precursors for Ga, In and N sources, respectively. The samples consist of a 2-μm-thick Si-doped GaN layer, a two-period unintentionally doped InGaN/(In)GaN MQW active region and a 150 nm Mg-doped GaN layer. The (In)GaN quantum barriers and InGaN quantum wells layers of three samples were grown at 750 °C, and a very thin GaN cap layer was inserted between the QW and QB layers to protect the QW layers from the etch effect of H_2_. Sample A is a reference sample and the (In)GaN barrier layers was immediately grown after the growth of the cap layer. For sample B, 100 (sccm) hydrogen flow rate was conducted after the growth of the cap layer and maintained 100 s. For sample C, 200 (sccm) hydrogen flow rate was conducted after the growth of the cap layer and maintained 100 s. During the introduction of H_2_, NH_3_ was still kept to introduce into the reaction chamber, whose flow rate was 3 slm for all samples. Thus, the latter two MQW samples B and C were treated in the H_2_/NH_3_ mixed gas during the hydrogen treatment process. Except of the above-mentioned difference, the growth conditions of the three samples were entirely identical.

The cross section images of MQW are examined via a JEOL JEM-F200 transmission electron microscopy (TEM). The structure properties of three samples are measured with a Rigaku SmartLab high-resolution X-ray diffractometer (HRXRD). Temperature-dependent (TD) photoluminescence (PL) spectra, which were recorded between 30 and 300 K, were measured using a He-Cd 325 nm laser in a closed-cycle helium refrigerator of CTI Cryogenics. Meanwhile, microscopic photoluminescence (μ-PL) with high spatial resolution was performed by using a Nikon A1 confocal optical system excited with a 405 nm laser.

## Results and Discussion

**Fig. 1:**
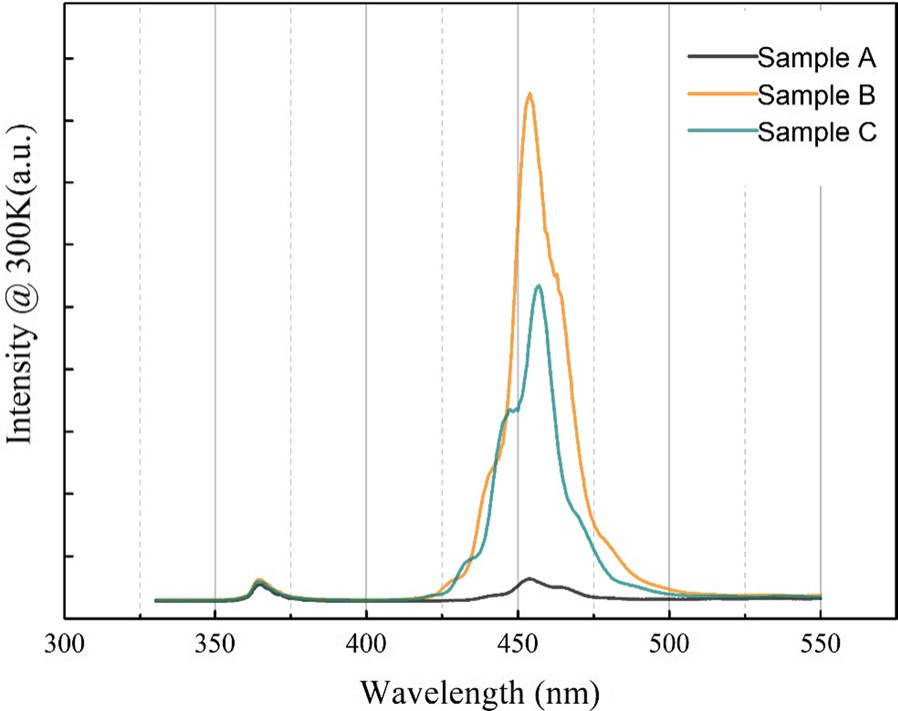
The PL spectra of three samples at 300 K. The luminescence intensity of sample B treated with 100sccm hydrogen is the strongest among the three samples

Figure 1 shows the PL spectra of three samples measured at room temperature (300 K). To compare the luminescent properties effectively, the PL spectra of three samples were measured under the same conditions, such as the slit width of spectrometer, the integration time and gain voltage of detector. It is noted that PL intensity of the main peak of sample B which is treated by 100sccm hydrogen is the highest among the three samples. The PL peak intensity of sample C is weaker than sample B, but stronger than sample A. The wavelength of main peak for all three samples is around 455 nm, which is correspondent with interband transition energy of InGaN/GaN MQWs. In the higher energy side, a small peak appears at around 365 nm, which corresponds to the near band gap luminescence of GaN. The results demonstrate that appropriate hydrogen flux (100 sccm) during the hydrogen treatment can significantly improve the luminescent performance, but the luminescent performance will deteriorate a little when using a too high hydrogen flux (200 sccm).

**Fig. 2:**
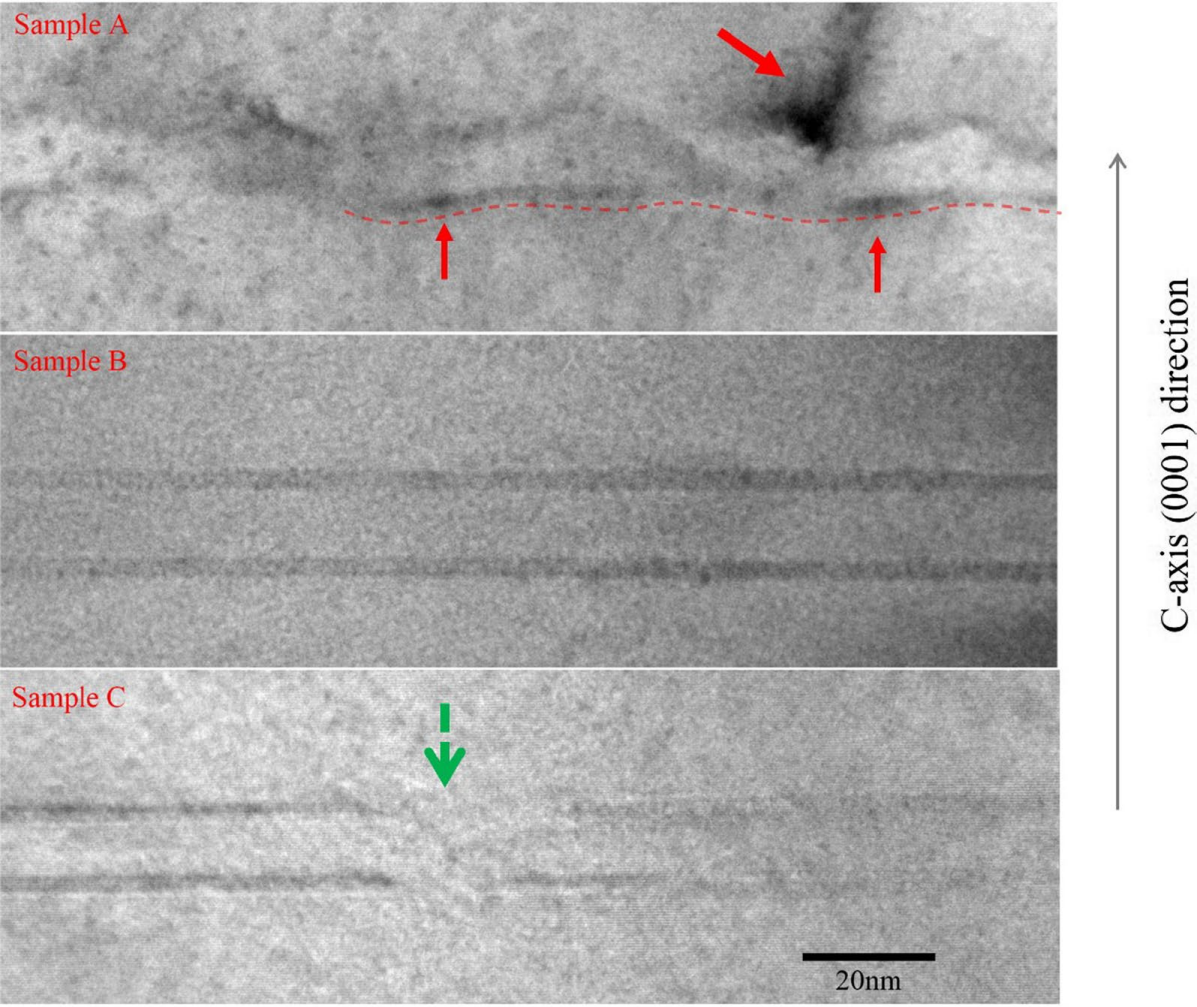
The cross section TEM images of samples A, B and C. In the image of sample A, the red dashed line marks the undulated MQWs interface and the red arrows point out the indium-rich clusters in MQWs. In the image of sample C, the green arrow points out the damaged part interfaces of MQWs

To investigate what happens to the MQW region during the hydrogen treatment, cross section transmission electron microscope (TEM) images of three samples are shown in Fig. 2. Three key points should be noticed in Fig. 2. First, the interfaces between QW layers and QB layers in sample A are undulated, and the QW thickness varies significantly as marked by red dashed line. But the interfaces of MQW in samples B and C are steep and flat, which can be distinguished in Fig. 2 clearly. The QW thickness fluctuation of sample B and C is small. Second, the distribution of atoms in the QW layers of sample A is not uniform but tends to aggregate near and on some places of the surface of the well layers, as shown by the red arrows. As the only difference between QB and QW layers is the indium content, the aggregated atoms should be caused by the indium segregation. Such phenomena actually had been observed in previous reports [11]. The atom’s aggregation is not found in sample B and sample C. Third, compared to sample B, the interfaces have disruptions in sample C as shown by the green arrow. In summary, when using 100 sccm flux for the hydrogen treatment, not only the interfaces of MQWs become steep and flat, but also the distribution of indium atoms becomes more uniform. However, when the hydrogen flux increases to 200 sccm, the disruptions appear in the interfaces again. It is noted that the uniformity of MQW layers as shown in sample B is normally able to achieve only after an temperature ramp-up process [19]. But here, the uniformity of the MQWs can be achieved at low temperature (750 °C) by hydrogen treatment process.

To understand the mechanism of the hydrogen treatment influencing the quality of MQW region, the reason for the worse MQW quality in sample A without hydrogen treatment should be figured out firstly. The appropriate deposition temperature of GaN is beyond 1000  °C at which the growth mode of GaN epitaxial layer tends to be step-flow mode [26, 27]. However, due to the low deposition temperature of InGaN/(In)GaN MQWs which now is as low as 750  °C, Ga atoms are hard to migrate to the edge of the steps due to the limited atomic surface mobility. As a result, the growth mode of GaN barrier layers tends to be 3D island-growth mode and the epitaxial layers are in a metastable thermodynamic state [28]. Thus, the surface is easily to be undulated as schematically shown by the red dashed line in Fig. 2. On the other hand, in the sample A, the indium atoms are found to aggregate as indium-rich clusters on the surface of QWs. Such behavior is mainly ascribed to the huge miscibility gap between GaN and InN, which is caused by the large mismatch between GaN and InN [15].

During the hydrogen treatment process, the ammonia (NH_3_) was still introduced into the reaction chamber. According to previous reports, the rate of surface coverage of NH_3_ content is relatively low (about 25%) and the main composition of the coverage is NH_2_ radicals (about 75%) [28, 29]. In such a low coverage of NH_3_, the binding energy of gallium (Ga)/indium (In) adatoms to the surface is relatively high, leading to a low surface-diffusion rate and weak desorption of adatoms [29, 30]. Because H_2_ is the product of ammonia decomposition, and the decomposition rate of NH_3_ decreases and the surface coverage of NH_3_ increases during the hydrogen treatment process, as a result, a weakened binding energy of gallium/indium adatoms caused by the increased coverage of NH_3_ enhances surface-diffusion rate and desorption of gallium/indium adatoms. Meanwhile, the hydrogen was introduced into the reaction chamber for 100 s, which allows gallium and indium adatoms to have a farther diffusion length. Therefore, the gallium and indium adatoms are more easily to achieve thermodynamic steady state, and the interfaces become flat and steep. Moreover, in the mixed gas environment of H_2_ and NH_3_, indium-rich clusters will desorb more easily than the indium-poor regions [31]. Thus, the distribution of indium content will be more uniformly across the whole well layers, resulting in a better MQWs’ homogeneity of sample B. However, when an excessive hydrogen flux (200 sccm) is introduced into the reaction chamber, the desorption of the indium adatoms will increase further and the QW layers are partly damaged due to the etching effect of hydrogen [32] as what is shown in the cross section TEM image of sample C in Fig. 2.

It should be noted that, Czernecki et al. reported that when the hydrogen treatment is conducted between the growth of barriers and well layers, the quantum wells will be etched and become undulated [28]. However, such kind of etching effect has not been observed in our work. It is assumed that there are two main reasons for the difference. Firstly, the hydrogen ions which will lead to etching effect are less due to the low temperature and the smaller amount of hydrogen flux. Secondly, before hydrogen treatment, a thin GaN cap layer was deposited on the QW layers, which can protect InGaN well layer from the etching effect. Therefore, in the mixed gas of NH_3_ and H_2_, the MQWs become uniform due to such kind of atomic rearrangement process.

**Fig. 3:**
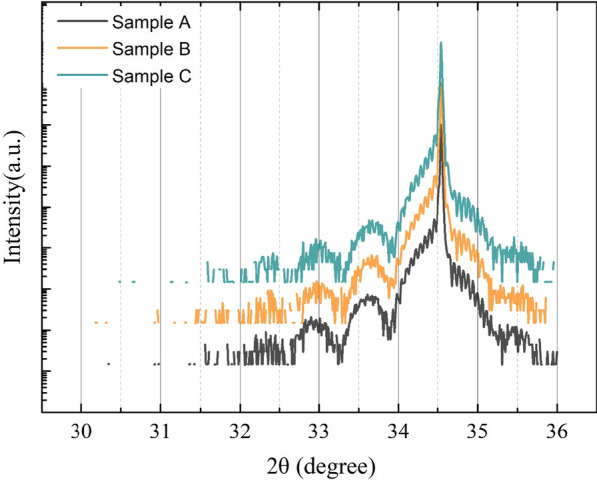
The ω-2θ scan curves on (0002) of sample A, B and C which are measured by high-resolution X-ray diffractometer (HRXRD)

As the scale of TEM images is in nanometers, the structure properties in a larger scale are investigated by Rigaku SmartLab high-resolution X-ray diffractometer (HRXRD). The ω-2θ scan curves on (0002) are shown in Fig. 3 and the parameters of InGaN/GaN MQWs are obtained by fitting the measured ω-2θ scan curves by using Global Fit program, as shown in Table 1. It is found that sample B has similar structure parameters to sample A except its relatively lower indium content of InGaN well layers. The decrease in indium content in QWs for sample B is mainly caused by etch effect of hydrogen. Moreover, not only the indium content, but also the thickness of QWs, are reduced evidently in sample C, which is caused by the overreaction effect of H_2_ treatment. It is also noted that the indium content and the thickness of QB layers of sample C increase obviously compared to samples A and B. It demonstrates that when the hydrogen flux is excessively too high during the hydrogen treatment, part of the desorbed indium atoms will incorporate into QBs, resulting a larger thickness and indium content of QB layers.

**Table 1:** Structural parameters of MQWs of samples A, B and C determined by HRXRD measurements

Samples	QW layers		QB layers	
	Thickness (nm)	In content (%)	Thickness (nm)	In content (%)
A	4.0	9.8	9.6	1.1
B	4.1	8.6	9.7	1.2
C	3.7	8.2	9.9	1.4

The effect of hydrogen treatment on the structure properties has been discussed in detail through TEM images and XRD. In the next several sections, how the changes of structure properties affect optical properties will be further studied.

**Fig. 4:**
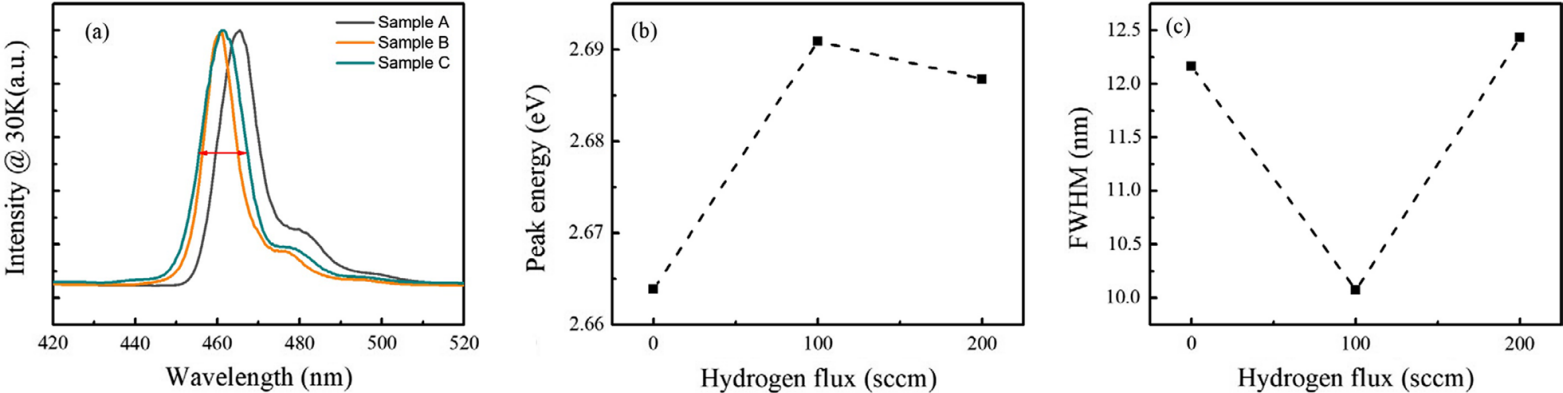
The PL spectra at 30 K (**a**); the peak energy (**b**) and FWHM (**c**) of sample A, B and C through gauss fit of the PL spectra

Figure 4 shows the PL spectra measured at 30 K and the comparison of some PL features of three samples. Due to the weak carriers’ transfer capability and the suppression of non-radiative centers at low temperature, the results of PL at 30 K are usually used to characterize the optical properties of radiative recombination centers in MQWs. In Fig. 4a, a side peak is obviously shown in the PL spectra for all the three samples. The energy gap between the side peak and main peak is around 90 meV, which is closed to the optical-phonon energy of GaN. Thus, it is safe to say that the side peaks are the phonon replica [33]. As shown in Fig. 4b, the peak energy of sample A is much lower than that of samples B and C, which is well corresponding to the results of HRXRD. But the peak energy of sample C is a bit lower than that of sample B, which may be caused by the poorer uniformity of MQWs of sample C. Figure 4c shows the full width at half magnitude (FWHM) of PL spectra at 30 K. The FWHM of the PL spectra of samples A, B and C is 12.3 nm, 10.1 nm and 12.6 nm, respectively, indicating that sample B has the best luminescence uniformity. Noted that the FWHM for sample C is at the same level as that of sample A, which means the discontinuity in the MQW’s interface severely deteriorates the uniformity of luminescence centers.

**Fig. 5:**
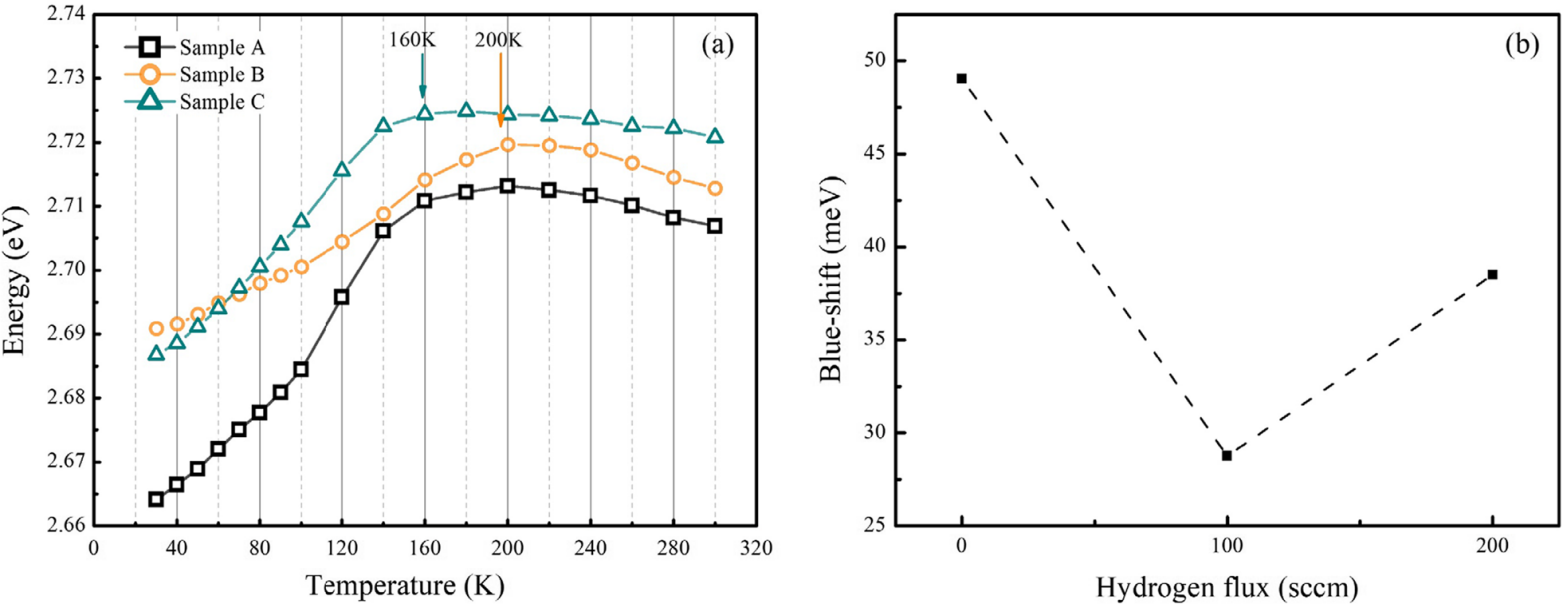
**a** The curves of peak energy versus temperature for samples A, B and C; the arrows indicate the red shift turning temperature. **b** The amount of blue shift of samples A, B and C; and sample B has a lowest amount of blue shift

To further check the luminescence properties of three samples, Fig. 5a shows the curves of peak energy of PL spectra versus temperature for three samples. The peak energy of all sample blue shifts first and then red shifts along with increasing temperature. As well known, in semiconductor materials, the peak energy will red-shift with increasing temperature due to band-gap shrinkage effect. But in GaN-based MQWs, a blue shift of peak energy with increasing temperature has been observed. Such a blue shift is caused by the different energy distribution of localized states in QWs. When the temperature increase, carriers transfer from deep localized states to shallow localized states. The energy position of latter’s is located higher [34, 35]. Thus, the larger the blue shift, the more inhomogeneous the distribution of localized states. Sample A has the largest blue shift, and sample B has the lowest blue shift among the three samples as shown in Fig. 5b, indicating that sample B has the most homogeneous distribution of localization states of MQW among the three samples. Combined with the result of TEM images in Fig. 2, two aspects lead to the inhomogeneity of sample A: the larger fluctuations of well thickness and the inhomogeneous indium composition. In addition, it is also noted that sample C has a different red shift turning temperature which is 160 K, while it is 200 K for sample A and sample B, indicating that excessive hydrogen flux during the treatment may introduce a new cause to lead to blue shift in sample C. Considering that the MQW interfaces are partly damaged by excessive hydrogen as shown in Fig. 2, it may be also a reason for the increase in blue shift. As reported in the literature, a redshift also often appears in the lower temperature stage [34], but it is not observed in this work. This is may be due to the lower potential barrier height which hinders the carrier transported form shallower traps to deeper traps.

**Fig. 6:**
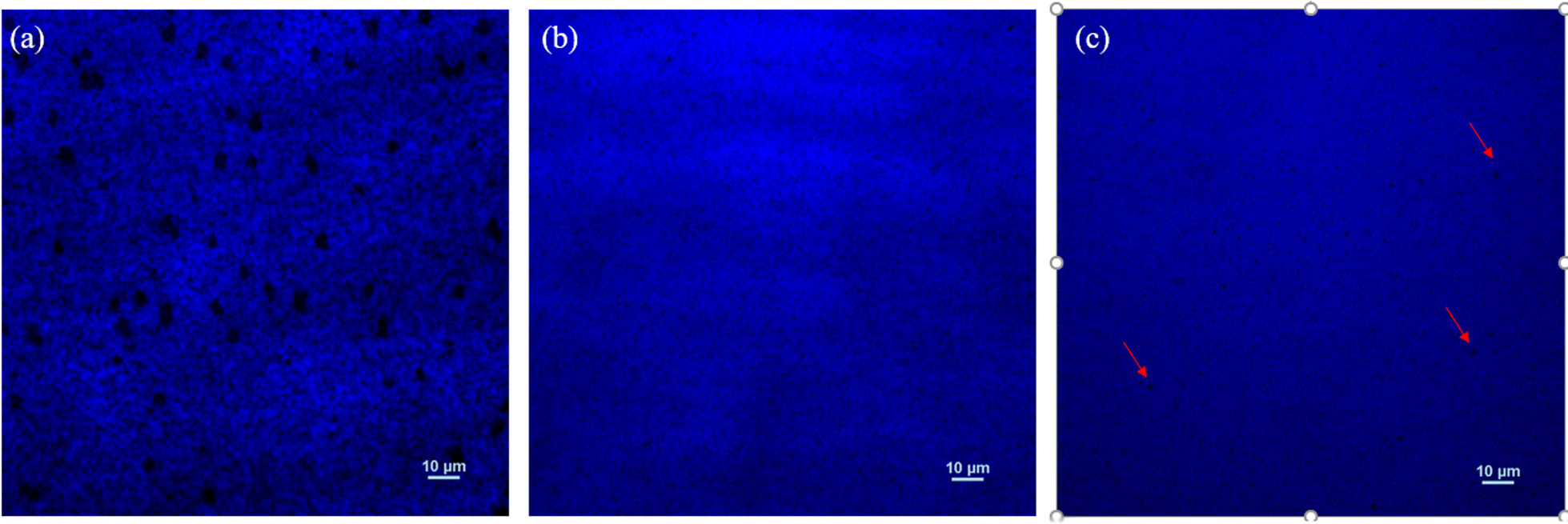
The comparison of micro-PL results for sample A, B and C which corresponds to (**a**), (**b**) and (**c**), respectively. The red arrows indicate the non-radiative luminescence region with smaller size in sample C

The limitation of TDPL is that it only characterizes the overall luminescence characteristics of the samples because it lacks the spatial resolution of emission properties. Therefore, the micro-PL of samples A, B and C is measured and shown in Fig. 6a–c, respectively. Significantly, the number and size of non-luminescence areas in sample A are the largest. After 100sccm hydrogen treatment process, the non-luminescence areas are clearly much reduced as shown in Fig. 6b. B. It is well known that indium atoms tend to accumulate around dislocation defects, resulting in a strong restriction effect on carriers around the area. During the hydrogen treatment process, a larger surface diffusion rate and the desorption process eliminate the aggregation of indium-rich clusters. Thus, sample B has less non-luminescence areas. However, when excessive hydrogen flux (200 sccm) was introduced into the reaction chamber, a few small non-luminescence areas reappear in the micro-PL image as pointed by the red arrows in Fig. 6c. It is mainly caused by the partly damaged MQWs regions as shown in Fig. 2.

Through above results and discussions, sample B has the most uniform luminescence properties and the least non-radiative recombination areas. These optical properties correspond to the strongest luminescence intensity in Fig. 1 very well. To further check the performance of the three samples, an approximation method for calculating the internal quantum efficiency (IQE) was presented. Take the internal quantum efficiency at 30 K as 100%, then the IQE at room temperature can be roughly calculated by the following expression:

1 $$IQE = \frac{{\mathop I\nolimits_{300K} }}{{\mathop I\nolimits_{30K} }}$$

**Fig. 7:**
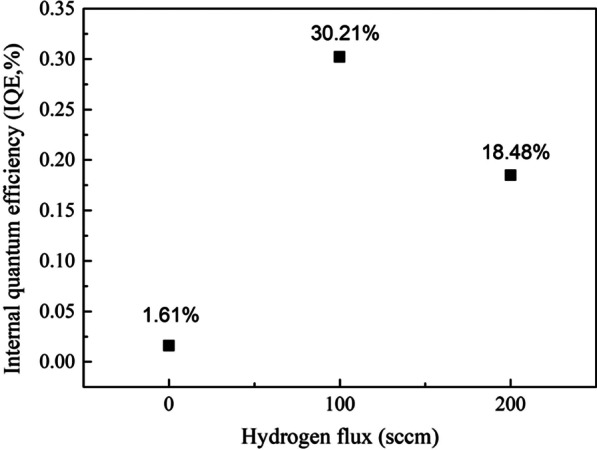
The internal quantum efficiency (IQE) of sample A, B and C. The IQE of sample B is as high as 30.21%

The *I*_*300K*_ represents the integrated intensity of PL spectra at 300 K and the *I*_*30K*_ represents the integrated intensity of PL spectra at 30 K. The results of IQE for three samples are shown in Fig. 7. The IQE increases strongly from 1.61 to 30.21% when using appropriate flux of hydrogen during the hydrogen treatments. The main reasons for the large increase in IQE of sample B are the better uniformity of both indium composition and thickness of MQWs and the reduced non-radiative recombination centers as discussed above. On the other side, when using excessive flux (200sccm) of hydrogen, the IQE decreases from 30.21% to 18.48% which is mainly caused by the partly damaged MQWs.

## Conclusion

In this work, GaN-based MQWs with better structural and optical properties grown at low temperature (750 °C) were achieved by using hydrogen treatment after the growth of InGaN well layers. Both sharp interfaces of MQW and homogeneous indium distribution are achieved when the hydrogen flow rate is taken as 100 sccm, which is caused by the appropriate atomic rearrangement of MQWs. Moreover, the luminescence efficiency is improved strongly due to the suppressed non-radiative recombination centers and a better homogeneity in MQWs. Such kind of atomic rearrangement process is main caused by the better diffusion rate of gallium and indium adatoms in H_2_/NH_3_ mixed gas, which leads to a lower potential barrier energy to reach the thermodynamic steady state. However, when excessively high hydrogen flux was introduced, the MQWs will be partly damaged, and the luminescence performance will deteriorate.

## Data Availability

The datasets used and/or analyzed during the current study are available from the corresponding author on reasonable request.

## Abbreviations

MQW: Multiple quantum well
LEDs: Light emitting diodes
LDs: Lasers diodes
MOCVD: Metal organic chemical vapor deposition
QB: Quantum barriers
TEM: Transmission Electron Microscope
HRXRD; TEGa: Triethylgallium
TMIn: Trimethylindium
NH3: Ammonia
H2: Hydrogen
TDPL: Temperature-dependent photoluminescence
μ-PL: Microscopic photoluminescence
FWHM: Full width at half magnitude
IQE: Internal quantum efficiency
